# *Clostridioides difficile*, a New “Superbug”

**DOI:** 10.3390/microorganisms11040845

**Published:** 2023-03-26

**Authors:** Rumyana Markovska, Georgi Dimitrov, Raina Gergova, Lyudmila Boyanova

**Affiliations:** Department of Medical Microbiology, Medical University of Sofia, 1431 Sofia, Bulgaria

**Keywords:** *Clostridium difficile*, superbug, virulence, mortality, epidemiology

## Abstract

*Clostridioides difficile* is a Gram-positive, spore-forming, anaerobic bacterium. The clinical features of *C. difficile* infections (CDIs) can vary, ranging from the asymptomatic carriage and mild self-limiting diarrhoea to severe and sometimes fatal pseudomembranous colitis. *C. difficile* infections (CDIs) are associated with disruption of the gut microbiota caused by antimicrobial agents. The infections are predominantly hospital-acquired, but in the last decades, the CDI patterns have changed. Their prevalence increased, and the proportion of community-acquired CDIs has also increased. This can be associated with the appearance of hypervirulent epidemic isolates of ribotype 027. The COVID-19 pandemic and the associated antibiotic overuse could additionally change the patterns of infections. Treatment of CDIs is a challenge, with only three appropriate antibiotics for use. The wide distribution of *C. difficile* spores in hospital environments, chronic persistence in some individuals, especially children, and the recent detection of *C. difficile* in domestic pets can furthermore worsen the situation. “Superbugs” are microorganisms that are both highly virulent and resistant to antibiotics. The aim of this review article is to characterise *C. difficile* as a new member of the “superbug” family. Due to its worldwide spread, the lack of many treatment options and the high rates of both recurrence and mortality, *C. difficile* has emerged as a major concern for the healthcare system.

## 1. Introduction

*Clostridioides difficile* is a Gram-positive, spore-forming, anaerobic bacterium in the form of a thick rod and sizes up to 4–5 micrometres. It was discovered in 1935 as part of the normal flora of healthy individuals and was named *Bacillus difficile* because of the problematic cultivation, and three years later was re-named *Clostridium difficile* [1]. After the implementation of antibiotics and their increased usage over time, the researchers made a connection between the microorganism and antibiotic-associated diarrhoea and pseudomembranous colitis [2]. Decades later, it was known as the most common cause of nosocomial diarrhoea, and in 2017 it was renamed to *Clostridioides difficile*, taking into account its differences with the *Clostridium* genus [3]. Taxonomically, *C. difficile* belongs to the order Eubacteriales, the family *Peptostreptococcaceae*, and the genus *Clostridioides. C. difficile* is peritrichous and motile and does not form a capsule, and it has a newly described protein S layer [4]. The S layer is a common structure for some bacteria and virtually all archaea. This structure is supposed to be associated with many activities, such as lysozyme resistance, bacteriophage adhesion and others. In *C. difficile*, the S-layer is composed of different proteins that form a two-dimensional structure [4]. 

The main pathogenic factor of *C. difficile* is the production of toxins [4,5]. *C. difficile* has been included in the list of microorganisms with an ‘urgent’ threat level by the Centres for Disease Control and Prevention [6]. During the COVID pandemic, with the enormous antibiotic usage and immunity problems caused by SARS-CoV2, the importance of *C. difficile* infections increased and needs urgent measures to solve the problem. Due to its worldwide spread, the lack of many treatment options and the high rates of both recurrence and mortality, *C. difficile* has emerged as a major concern for the healthcare system, and the aim of this review is to explore the possibility of designating this microorganism as a new member of the ‘superbug’ family.

## 2. Material and Methods

A literature review was conducted in January 2023 using Pubmed and Scopus databases. The search criterion was “*Clostridioides difficile*”. More than a quarter of the reviewed papers were from the period 2020–2023; another quarter was for the period 2015–2019. In Bulgaria, 78 *C. difficile* isolates were observed in the Department of Medical Microbiology, Sofia, between 2012 and 2022. They were tested for the gene encoding glutamate dehydrogenase and for *tcdA/tcdB* and *cdtA/B* with PCR, as previously described [7]. 

## 3. Discussion

### 3.1. Factors of Virulence and Pathogenesis

*C. difficile* produces different exotoxins as its main pathogenic factors. The two major toxins are TcdA of 308 kDa and TcdB of 270 kDa in size. Both toxins have enterotoxic and cytotoxic effects, but in the past, TcdA was regarded as enterotoxin A and TcdB as cytotoxin B [8,9]. Both toxins are glycosyltransferases (GTPases) that inactivate human Rho GTPases. They increase the production of proinflammatory cytokines such as IL-1, TNFα, and IL-8, which lead to leukocyte infiltration, increased inflammation and fluid secretion; they also cause depolymerisation of the actin filaments, disruption of the tight junction and loss of intestinal barrier function, cell apoptosis, and gut epithelium damage. In severe cases, thick yellow fibrous exudates, called pseudomembranes, are formed. They are made of leukocytes, macrophages, and necrotic epithelial cells and are a main finding in pseudomembranous colitis [9,10].

Both toxins are identical in 47% of their structure and are in the group of ABCD toxins (A—activity (glycosyltransferase domain), B—binding (receptor binding domain), C—cutting (cysteine protease domain), D—delivery (delivery and receptor binding domain) with the domains being in the following order: first is the A domain in the N end of the molecule and is a Rho glycosyltransferase. Next is the C domain—a cysteine protease that initiates the autoactivation of the toxins, followed by the D domain, which is responsible for pore forming and the delivery of the toxin into the cytosol of gut enterocytes. At the other end (C end) of the molecule is the B domain—responsible for receptor binding. The toxins enter into the cells by receptor-binding endocytosis; the receptors for the TcdA and TcdB are different. In the endosome, they cause acidification which activates and causes pore formation. This is followed by the release of the (glycosyltransferase) domain with the help of cysteine protease, which acts as an activator. Glycosyltransferase targets the Rho proteins, which have many functions, such as regulating the function of the cytoskeleton and cell communication. The glycosyltransferase inactivates those proteins, thus blocking the cell signalisation, which leads to cytopathic effects. This results in morphologic changes such as rounding of the cell, destruction of the cytoskeleton and tight cell-to-cell contacts, increased permeability and ultimately, diarrhoea [9,10,11]. The cytotoxic effects are mainly associated with inflammation and activated apoptosis [10,12]. Although the toxins are similar, they appear to have different mechanisms of action. Due to experiments with animal tissues, it was previously thought that toxin A was more potent, but similar tests with human tissues showed that toxin B is the more active one. Strains that produce only toxin B were found to cause disease and even death in humans. Further experiments have shown that these strains produce a modified toxin B that has an activity similar to that of toxin A without the presence of toxin A. Those experiments and the fact that a monoclonal antibody against toxin B is used in CDI treatment imply that toxin B is of higher importance than toxin A [9,13,14].

The genes encoding the toxins are in a pathogenicity locus called PaLoc, which is 19.6 kbp and is located in the chromosome. Non-toxigenic strains do not have that locus but can gain it through conjugation. In the PaLoc, there are five genes in total: *tcdA*, *tcdB*, *tcdC*, *tcdE*, and *tcdR. TcdA* and *tcdB* encode toxin A and toxin B, *tcdR* activates the expression of *tcdA* and *tcdB* and its own expression, and the latest data show that *tcdC* acts as a negative regulator [10,15,16]. The synthesis of tcdR and the following expression of *tcdA* and *tcdB* is influenced by many factors, such as the presence of short-chain fatty acids, sub-inhibitory concentrations of antibiotics and others [13]. 

In the last decades, new strains, marked as “hypervirulent”, have emerged. They cause severe *C. difficile* infections (CDIs), often without previous antibiotic usage and disruption of the gut microbiome. They produce a large amount of toxins, TcdA and TcdB, due to mutations in *tcdC* (18 bp deletion) and an additional (third) toxin—the binary toxin Cdt (*C. difficile* transferase). Cdt is composed of *cdtA* (enzyme component, ADP ribosyltransferase) and *cdtB* (binding component, which allows binding, entry of the toxin, endosome creation and delivery into the cytosol). *cdtA* damages the cytoskeleton of the enterocytes, causes a redistribution of the microtubule network and leads to cell rounding, fluid loss, and cell death. *cdtA* and *cdtB* genes are located in a different locus (CdtLoc 6.8 kbp) in the chromosome, and *cdtR* positively regulates their expression. There are two variants of *cdtR*, one is truncated *cdtR*, and the other has a 68 bp deletion; in both cases, the levels of secreted toxins decrease. The exact role of CDT is not clear, and the reported data shows that only CDT is usually not enough to cause a disease, but in combination with the high amount of TcdA/B toxins, it can cause a severe disease [17,18,19]. The complexity of the problem is shown by the fact that there are rare cases of *tcdA^−^tcdB^−^* and *cdtA/B* positive isolates that can cause symptomatic CDI. A recent study describes low-toxin RT027 strains that exhibited similar to high-toxin RT027 strain lethality in hamster models. This shows the need for reconsidering the clinical significance of a TcdA/B negative result and underscores the potential limitations of current diagnostic protocols [20]. Interestingly, in contrast a study reported that the virulence of RT027 strains does not exceed the virulence of other ribotypes [21]. 

The *C. difficile* BI/NAP1/027 first appeared in Europe and North America early in the 21st century and was named “hypervirulent” due to its ability to cause infections without previous disruption of gut microbiota and more severe cases of CDIs. It displays high amounts of TcdA/TcdB toxins due to the inactivation of *tcdC* by an 18 bp deletion and production of binary toxin. Another commonly detected hypervirulent strain belonged to ribotype 078 [9,17,22].

In addition to the above-mentioned toxins, *C. difficile* produces many enzymes such as collagenase, chondroitin-sulfatase, and hyaluronidase that also cause disruption of tight junctions and fluid loss [9,13].

One additional factor of virulence is surface protein, a two-dimensional structure named S layer. Interestingly, *C. difficile* isolates without S layers showed decreased virulence, decreased resistance to lysozyme and an inability to produce symptoms of CDI in hamsters. They also showed reduced spore formation and spores varying in heat resistance. The investigation of the S layer may give a new important target for the development of antibodies [4,5].

Sporulation may not be the main virulence factor, but it is still a challenging and very important step in CDI. The spores are resilient and have the ability to stick to the walls of the colon. Spores may play a major role in the recurrence, and patients can be a source of infections for up to 4 weeks after recovery. This can explain the wide distribution of the spores into the environment. The spores are reservoirs for survival for the *C. difficile* cells and can explain the recurrent infections as antibiotics cannot enter through the spore layers [10]. 

Factors associated with the germination of the spores are very important. Once ingested, spores can survive in the stomach and thereafter in the duodenum and begin to germinate back to vegetative cells. Germination is initiated in response to germinants, chemical signals that activate the spores to revert into vegetative cells. The major germinants for *C. difficile* are the primary bile acids and mainly taurocholate [10]. 

One important factor of pathogenicity is biofilm production, which has recently been investigated. It forms part of the healthy, multi-species biofilm during asymptomatic carriage. Biofilm production can influence the immune response and plays an important role in recurrence. Its regulation is not fully understood [23,24].

### 3.2. C. difficile Infections (CDIs) and Associated Risk Factors

*C. difficile* infections can appear as asymptomatic infections and mild diarrhoea to haemorrhagic diarrhoea and pseudomembranous colitis. CDIs can affect the colon, especially the distal segment and are the leading cause of antibiotic-associated diarrhoea [9,13]. 

The definition of CDI used the following criteria proposed by the European Centre for Disease Prevention and Control (ECDC): diarrheal stool (≥3 times per day for more than 24 h) or toxic megacolon and positive laboratory tests for TcdA and/or toxin B [25]. 

Watery diarrhoea is the main symptom of mild or moderate CDI. Other manifestations include mild abdominal pain or cramping, low-grade fever, and nausea [26]. Fever is a rare symptom in no more than 15% of mild cases. Mild CDI is commonly associated with moderate leucocytosis—white blood cell count can be up to 15,000 cells/mL [26]. Some patients with mild CDI can recover spontaneously after 5–10 days after the end of the antibiotic therapy. However, in some cases, the antibiotics cannot be withdrawn. 

A specific case was the asymptomatic carriage of *C. difficile* after recovery; patients can carry *C. difficile* for around a month [9,17]. Children aged less than 2 years can also carry *C. difficile* without symptoms since they do not have receptors for the toxins [17]. Asymptomatic carriage is one of the main drivers of *C. difficile* dissemination. 

In some patients, CDI appears as severe haemorrhagic colitis with haemorrhagic or watery diarrhoea (10–15 times per day), diffuse severe abdominal pain, fever, elevated creatinine concentration and leucocytosis (white blood cell count 20,000–40,000 cells/microL or higher). Endoscopic investigation can reveal specific changes in the colon endothelium (pseudomembranes and ulcers), which gives the name of the disease pseudomembranous colitis [25,27]. Fulminant colitis can cause severe hypotension and multisystem organ failure, and renal insufficiency. Shock, ileus, or megacolon can be complications of fulminant colitis [25,27]. Extracolonic manifestations of CDI are rare and most commonly involve reactive arthritis and bacteraemia [27].

Recurrent CDI is defined as a reappearance of symptoms within two to eight weeks after appropriate treatment has been stopped [25,27]. The cause of recurrence is not completely understood, but researchers have some hypotheses. The main of them is the prolonged disturbance of the gut microbiota by treatment, defective host immune response, and persistence of the spores. Up to 25–30% of patients can have recurrent CDI within 30 days after the treatment ends [28,29]. Recurrent symptoms may be due to relapse of the initial infecting strain or reinfection with a completely new strain. Less commonly, recurrent CDI can occur as late as two months after discontinuation of treatment. Once patients have experienced one recurrence, they are at significantly increased risk (40–65%) for further recurrences [10,13,28].

Risk factors for CDIs are mainly associated with previous hospitalisation and antibiotic treatment. The prolonged length of hospital stays and frequent hospitalisations are the main risk factors. *C. difficile* sporulates in the colon, and the spores are extremely resistant in the environment to drying, temperature, and many disinfectants. Spores can persist on environmental surfaces for months, and patients can be infected directly or by the hands of personnel or from other patients who carry *C. difficile* [30,31]. The reservoirs are convalescent patients and healthy carriers. 

In the early stages of human life, the prevalence of *C. difficile* is the highest (15–70%) [32,33]. It is notable that small children are rarely symptomatic due to the lack of toxin receptors and the presence of protective antibodies in the mother’s milk [34]. *C. difficile* carriage can be found in 2.4–17.5% of healthy adult people in the community [33,35,36], but in hospitals, the carriage of *C. difficile* is much higher, showing a big threat of infections. The colonisation was between 2.1–20% during the first days of hospitalisation and increased up to 45–50% one month later [9]. This data shows a wide dissemination of *C. difficile* spores resulting in easy infection and increases the importance of the next risk factor—antibiotic usage. 

The use of antibiotics is one of the most important factors for the development of CDIs. The antibiotics that carry the highest risk of CDIs are clindamycin, fluoroquinolones, cephalosporins and penicillins, but the use of any antibiotic can predispose to CDI [10]. Paradoxically, antibiotics that are the main treatment options for different infections are also a main risk factor for *C. difficile* infections. Not only the type of antibiotic is important, but the dose, the use of combined therapy and increased duration are also important as factors for CDIs [9]. The risk for the development of CDI is 8- to 10-fold higher during antimicrobial therapy and around three months after discontinuing the therapy, with a higher risk in the first month [36]. The antibiotics can disrupt the balance of normal gut microbiota and inhibit some bacterial communities, followed by *a* loss of diversity and “colonisation resistance” in the gut. The most represented phyla in the gut are *Firmicutes* (64%) and *Bacteroidetes* (23%), followed by *Proteobacteria* (8%), *Fusobacteria*, *Verrucomicrobia*, and *Actinobacteria* (3%) [37]. A healthy asymptomatic carrier has no significant difference in gut microbiome compared to a non-carrier. The commensal flora in such subjects could protect the host by preventing the overgrowing of *C. difficile* and the production of toxins. In asymptomatic *C. difficile* gut carriers, antibiotic administration is followed by changes in bile metabolism and spore germination. Germination of spores is activated by primary bile acids from the liver, such as taurocholate and other cholic acid derivates, by the germinant receptor CspC and is inhibited by secondary bile acids in the colon (such as chenodeoxycholic acid). Glycine can also act as a germinant through an uncharacterised mechanism [13]. The other authors showed that butyrate concentrations are lower in faecal samples from humans with CDIs than in those from healthy controls [38]. This can give some possibility for dietary interventions. In addition, antibiotic administration can decrease the number of butyrate-producing bacteria (*Faecalibacterium prausnitzii*, *Eubacterium* spp, *Roseburia* spp.), thus increasing inflammation and *C. difficile* spore germination [39]. The microbiome changes facilitate the colonisation, germination of the spores and overgrowth of *C. difficile*, production of toxins and disease [40]. Against CDI, protective species are *Bifidobacteria* and some *Firmicutes* and the increase in *Proteobacteria* is associated with an increased risk of CDIs [39,40]. This can explain why some diseases, such as inflammatory bowel disease (IBD), can be a risk factor for CDI. Patients suffering from IBD showed a decreased diversity of *Firmicutes* and *Bacteroidetes* and the presence of pathogenic bacteria from the *Proteobacteria* phylum [41]. Other reports suppose that the presence of *Porphyromonadaceae*, *Lachnospiraceae*, *Lactobacillus*, and *Alistipes* might inhibit the growth of *C. difficile* [37].

Another important point is the fact that different antibiotics cause different changes in the microbiome. For example, some recent investigations showed that gut microbiome disbalance after quinolone (ciprofloxacin) usage could last for around 3 months, and the effect of clindamycin can last for around 1 to 2 years, with bigger decreases in *Bacteroidetes* in cases of clindamycin usage [39]. This can explain why different antibiotics can have different impacts on the risk of CDI. The increased usage of antibiotics in the recent COVID pandemic will worsen the situation. The other point of view is the fact that some community-acquired CDIs (CA-CDIs) started without previous antibiotic usage—about 30% of patients with CA-CDIs had not been exposed to antibiotics, according to previous reports [42]. This is associated mainly with the appearance and distribution of hypervirulent 027 isolates of *C. difficile*. 

**Figure 1:**
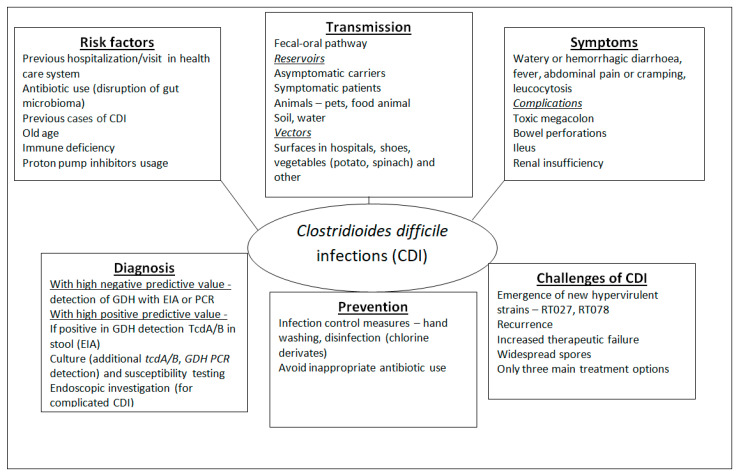
Main characteristics of *Clostridioides difficile* infections [9,10,17]. Abbreviations: EIA—enzyme immunoassay; GDH—glutamate dehydrogenase.

Old age is also an important factor (patients over 65 years have a 10× higher risk of developing *C. difficile* infections) [43]. Other factors that can contribute are proton pump inhibitors (PPI) usage, immunodeficiency, underlying diseases and most importantly, previous cases of CDI [29,44]. Most of the studies found the risk of CDI to be between 1.75 and 2.75-fold higher among those people that take PPIs [45]. Some studies explain connections between these factors; for example, patients taking PPIs showed significant increases in *Enterococcus*, *Streptococcus*, and *Staphylococcus* spp. in the gut [46]. The main risk factors are summarised in Figure 1. 

### 3.3. Epidemiology

The epidemiology of CDI is very interesting. Some characteristics of CDIs are shown in Figure 1. The first documented case was published in 1892—a patient, a 22-year-old female, who had bloody diarrhoea after surgery— and the case was reported by Finney et al. [47]. Interestingly, it had not been associated with antibiotic treatment, but the patient had boric acid stomach irrigation. Later, there was a reported strong association between pseudomembranous colitis and antibiotic usage, mainly of clindamycin [2,9,10]. Until the end of the 20th century, CDIs were accepted as a complication of antimicrobial therapy, were mainly hospital-acquired and were not accepted as a major problem for the healthcare system [9,10,43]. 

Thereafter, CDIs changed; they appeared more frequently, involving the community (antibiotic-naïve people, peripartum women and children), the isolates were more virulent (hypervirulent *C. difficile* 027 and less often 078 ribotypes), causing outbreaks, and the frequency of recurrent infections increased. In the first years of the 21st century, the rate of CDIs increased in North America and Europe. The infection rates showed a 47% increase in the period 2006–2010 compared to that between 2000–2005 in the USA [17,43]. The investigation of the overall incidence of CDI in the USA during the 1991–2005 period showed a big increase [48,49]. This increase was observed not only in the USA but also in Canada [50,51] and some European countries [13,52]. Some authors suggested that the increase in the frequency of CDI was due to epidemic distribution. Large outbreaks happened in the USA, Canada and the UK, peaking in 2004–2006 [9]. The epidemic in Europe, mainly by the hypervirulent 027 *C. difficile* strain, started with Holland, Sweden and England, then Belgium, Germany, Austria, and France have been involved and finally reached Greece and Spain [52]. 

The increase was also detected in 2009 in the USA when the total admissions associated with CDI accounted for 1% of all admissions [43]. In the USA, almost half a million people were diagnosed with symptomatic CDIs and were treated at the cost of over 6 billion US dollars. The continuous increase in CDI incidence was not common for all the countries during the next periods. In England, from 2007 to 2010, there was a 61% reduction in CDI incidence. This reduction could be due to the successful implementation of prevention and control policies [43]. In the United States, a decrease from 476,400 cases in 2011 to 462,100 in 2017 for the total amount of CDIs, was observed. This was on the basis of a decrease in HA-CDI because, for CA-CDIs, an increase from 170,000 cases in 2011–2012 to 462,100 in 2017 was observed [43]. In Canada, the researchers also detected a decrease in total CDI frequency between 2009 and 2015, with a relative increase in CA-CDI [53]. A US survey showed the changes in the HA- and CA-CDI percentages between 2011 and 2017. In 2011 from over 15,000 cases, 65% were hospital-acquired, and 35% were community-acquired. In 2017 the corresponding percentages were 51% and 49% [17]. The same happened across Europe—the survey in 2013 covering 14 countries showed HA-CDI prevalence between 44% (UK-Scotland) and 94% (Poland) [54,55]. The prevalent ribotype in these studies was ribotype 027. According to the ECDC guidelines in 2018, CDI cases are categorised as hospital-acquired when diarrhoea onset happens >48 h after admission and <1 month after discharge from a medical care facility, and community-acquired infection is when diarrhoea onset happens within 48 h after admission and if there is no hospitalisation within the previous 3 months [25]. A more recent survey, from 2016–2017, started in Europe (European hospital-based surveillance of CDI) revealed that almost 200,000 cases were reported annually [56]. Hospital-acquired (HA) CDIs in Europe for the period 2016–2017 were 60.9%, and CA-CDIs were 32.7% [56]. In this surveillance (2016), the most common ribotypes were RT014/020 (clade 1) with 16.8% and the hypervirulent RT078 (clade 5) with 7–11% (predominant is Czechia, Ireland and the Netherlands). The third most common ribotype was RT027 which prevailed in some European countries such as Hungary (67.6%), Poland (63%), Slovenia (44%) [56,57,58], and Serbia (27%) [59]. This data shows a wide dissemination of RT027 lineage [56]. Other important RTs 027like (ST1) types are RT176, RT036/198, RT016 and RT181 [56,60]. RT 176 was a common ribotype detected in the Czech Republic and was in second place in Slovakia and Poland [59,60]. In Serbia and Slovakia, the prevailing ribotype was 001 [60]. The longitudinal study performed by Freeman et al. for the period 2011–2016 across Europe showed similar results—RT027 was the predominant RT for five years, followed by RT001 in 2011 and RT014 in 2012–2014. In 2015, RT106 and RT002 were detected as well [61]. Wide dissemination of hypervirulent RT027, 027-like and 078 ribotypes showed the increasing threat to the health care system. 

Recently introduced multilocus sequence typing more correctly defined the clonal relatedness between isolates than ribotyping, but the problem is that there is no strict association between ribotypes and ST types. One ribotype can include one to three different ST types, and conversely, one ST type can include more ribotypes. In this light, many authors investigated ST and ribotypes. For example, ST1 includes RT027, RT176 and some other *cdt*-positive strains. In Denmark (2016–2019), the dominant RT was 014/020 (ST2/13) in around 19.5% of the cases. The authors detected that ST1 (RT027) accounted for 10.8%, with a temporal decline in the investigated period, and also detected ST11 (RT078) in 6.7%, ST8 (RT002) 6.6%) and ST6 (RT005/117) in 5.1%. The data showed a temporal decline of ST1 and an increase in ST103, ST17 and ST37 [62]. Another country which reported a very high frequency of Healthcare Associated CDI was Germany—92.1% of all CDI cases. It is not surprising that the prevalent ribotype was RT027 in 31.3%. RT014 took second place [63]. In Greece, the dominant RT types were RT181, RT126 and RT78 (all of them from *cdtA/B* positive linage) [64].

In Bulgaria, seventy-eight isolates positive for glutamate dehydrogenase were observed in the period 2012–2022 in the Department of Medical Microbiology, Sofia. From them, 59 (75.6%) were toxigenic, the main genotypes being *tcdA*/+/*tcdB*/+/ in 58 isolates and *tcdA*/−/*tcdB*/+/ in one isolate. A large number of strains (33.9%, 20 isolates) had *cdtA/cdtB* genes and were determined as hypervirulent. There were two such isolates for the period 2012–2017 and 18 for the period 2018–2022, which reveals a big increase in hypervirulent strains, similar to that in Poland, Check Republic, Germany and other countries [56,57,58,60,61,63]. Interestingly, in Bulgaria, the main types previously reported in 2013 were RT017 and RT014/020 [65]. 

**Figure 2:**
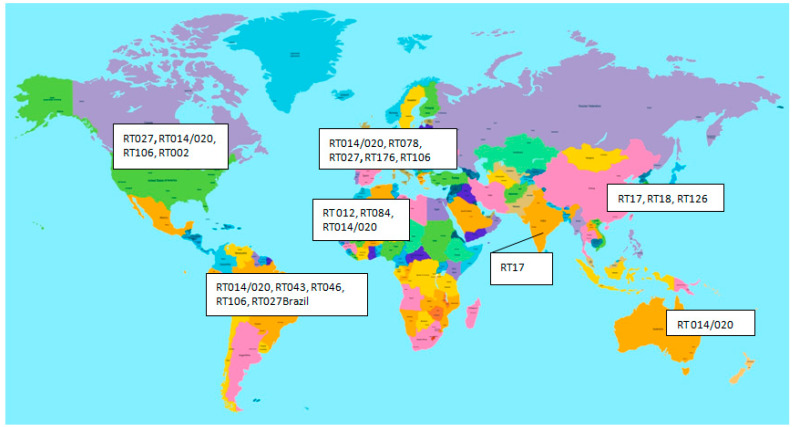
Distribution of some important *C. difficile* ribotypes in different continents [17,57,58,59,60,61,62,63,64,65,66].

The distribution of the ribotypes and the incidence were different in the other continents. In Asia, RT027 and RT078 rarely occurred, and the pooled CDI prevalence was lower than those in America and Europe—12.4%, with the prevalent ribotypes RT001, 002 and 012. The Far East is characterised by the appearance of Tcd(A-B+) strains of RT017. RT027 predominance is reported for Israel and Iran [66]. In Japan, the binary toxin-positive isolates (RT027 and 078) were rare (2–6%), with one exception, the RT027 outbreak in 2019. Interestingly, these isolates were moxifloxacin susceptible, suggesting that Japanese RT027 represents the pre-epidemic RT027 genetic background [67]. Data from Africa show that CDI patient populations are often younger than those in Europe, probably due to the high prevalence of co-morbid conditions such as tuberculosis, particularly in sub-Saharan Africa, the main ribotypes are RT012, RT084 and RT014/020 [68]. Another country that reported the prevalence of ST37 (RT017, clade4) is China. Another main ST is of clade 1 (ST3, ST54 and ST35) [69]. In Latin America, the authors reported similar findings as in other countries (Middle East, Asia, and China), with CDI incidence of around 19% and rarely detected RT027 with the exception of Brazil [70]. In the USA, there was a reported decrease in RT027 strains, but this ribotype was still prevalent. Other observed ST types were RT014/020, RT106 and RT002 [17]. In Australia, the main type is RT014/020 [42]. The distribution of some important RT types is shown in Figure 2. 

The recurrence of the disease was also high—with 20–25% of the cases [10]. The level of recurrence (re-infection of relapse) has also increased in recent years, up to around 30% [10]. 

Therefore, it is not strange that CDIs were one of the main causes of diarrhoea-associated death. *C. difficile* mortality increased from 10 to 48 per 100,000 person/year if comparing two periods, 1999–2000 and 2006–2007, as it was reported by some authors [71]. A similar study from Canada, for the similar period 1997/2005, revealed a four-fold higher mortality rate in 2005 than in 1997. Studies in England and Wales showed that the death rate caused by *C. difficile* increased from 975 in 1999 to 2247 in 2004 [44]. Most probably, the increased mortality in the 21st century was associated with the appearance of epidemic clones ribotype 027 and 078 that produce binary toxin and cause more severe diseases. Increased recurrence rates also affected mortality rates. In Germany, the recurrence rate within 30 and 90 days after CDI diagnosis was 15.7% and 18.6%, and the mortality rates were 21.4% and 41.4%, respectively [63]. ECDC surveillance (2016) detected that 4.1% of deaths are “possibly” related to CDIs [56]. In other studies, mortality within 1 month of diagnosis reached 9% of all cases, showing the high importance of this pathogen [17]. 

In the seventies of the previous century, it was accepted that the most important pathway for the transmission of *C. difficile* is the faecal-oral pathway and hospital settings with the wide dissemination of *C. difficile* spores in them. The most important introducers of the spores are asymptomatic carriers. The patients and the personnel have been infected directly by patient-patient or personnel-patient contacts or by the shedding of spores into the hospital environment. The asymptomatic carriers of toxigenic strains are more vulnerable to CDI. The opposite applies to carriers of non-toxigenic strains. In this case, they are protected from CDIs [13,17]. With the change in CDIs from the start of the 21st century and the increase in CA-CDIs, the sources could be outside the hospital setting. Food animals, companion animals and the environment play a critical, still underappreciated role in *C. difficile* transmission to humans. There is data that *C. difficile* spores can also persist in the food chain [71,72].

*C. difficile* can be found worldwide in the environment and in the gut of animals, companion animals (dogs and cats) or food animals. The pets can carry and be a source of *C. difficile*, regardless if they have diarrhoea or are asymptomatic [73]. They live in close and long contact with their owners, and the infection can easily spread in both directions. 

There are surveys indicating *C. difficile* can be found often in companion animals without intestinal symptoms. Adult dogs can carry *C. difficile* and often are asymptomatic. However, there is a reported case of a cat vomiting, in which an autopsy revealed severe necrotising haemorrhagic enterocolitis, and the authors supposed this cat to have a CDI [74]. Toxigenic strains have been detected in fewer than 20% of healthy and diarrheic dogs [73]. In 90% OR percents of puppies, similarly to humans, *C. difficile* was isolated at least once in the first 10 weeks. After 3 months of age, carriage dropped significantly [75]. It is important to note that the clinical features of CDI in animals can be really different [73]. The dysbiosis of the gut seems to play a far lesser role for dogs than for humans, and even more, in a state of dysbiosis, dogs tend to show symptoms of growth of other bacterial species [76,77]. This suggests a possible resistance of dogs against CDI. Comparative microbiome analysis shows that one of the changes in gut dysbiosis in dogs is an increase in the abundance of *Firmicutes*, whereas, in humans with gut dysbiosis, there is a decrease in the abundance of *Firmicutes* [78]. It is notable that members of this phylum can convert primary to secondary bile acids, and in humans, the increase in the latter creates a bile acid profile associated with CDI resistance [79]. Since CDIs in animals are often asymptomatic, they can be silent reservoirs for toxic and even maybe resistant *C. difficile* strains. Interestingly, the cats carried more toxigenic strains than dogs [80].

It is also important to note that the interaction between humans and different animals (time spent in close contact) could also play a big role in potential transmission. A British survey showed a significant correlation between the colonisation rates of infants and the presence of dogs in the same household [81]. Another survey from Canada showed a 26.7% asymptomatic carriage in dogs and cats, and authors proved the household transmission between domestic pets and their owner on the basis of an epidemiological relationship between isolates detected by pulsed-field gel electrophoresis (although the discriminatory power is not the same as whole genome sequencing) [82]. The role of dogs as support animals can also be an important factor. In 2006, a pathogenic human strain was identified in a dog that was visiting patients in hospitals [83]. A later study showed that support dogs used in hospital settings could be at 2.4 times more risk of acquiring CDIs than those used in other animal-assisted activities [83]. In even more recent studies, researchers isolated toxigenic CD strains from the nasal secretions of dogs [84] and from unprotected sandboxes [85]. They could be considered as a new vector for transmission. In addition, it is also important to mention the mechanical dissemination of *C. difficile* spores from paws and shoes [86]. 

Evidence that pets can play an important role in *C. difficile* dissemination is the fact that in companion animals, the researchers detected similar RTs as in humans. The isolates of ribotype 106, RT078 and 014/020 have been isolated from humans and also from cats and dogs [73,80,87,88]. A bigger challenge may be the fact that some of the isolated strains have shown resistance to metronidazole [78,89,90], which could pose a serious threat to human health.

Apart from pets, other possible factors for *C. difficile* transmission are animal farms. RT078 is a primary animal strain, and early in the 21st century, it was isolated from different animals [8,9,73]. Then it infected humans and caused outbreaks in Canada, the USA and Europe. There is a concern that there might be a connection between the rising frequency of isolation of *C. difficile* from food animals and community-acquired CDIs. However, this is quite questionable due to the lack of enough information and evidence for whether direct transmission is possible in light of the low prevalence of *C. difficile* in animal-derived foods [73]. That is why the findings of Knetsch C et al. [91] are very interesting, proving by whole genome sequencing that identical RT078 isolates (with two nucleotides difference in the genomes) were observed in farmers and farm animals [91]. Ribotype 078 is commonly found in pigs [92,93] and other food animals [94]. However, the frequency of *C. difficile* in raw meat was low (1.6%) in a Canadian study [95]. This problem still needs more investigation.

Due to the wide distribution of *C. difficile* and its ability to produce spores, more animal species pose a risk of infection via the faecal-oral route. Different strains have been isolated from horses, some of which are associated with human outbreaks (RT014, RT078) [96]. Researchers also found shared antibiotic resistance genes between equine and human strains [97]. That contributes to the possibility of interspecies transmission or adaptation of different toxigenic *C. difficile* strains [98,99]. 

Due to the ability of *C. difficile* to survive in the environment for a long time, researchers have looked for additional vectors of transmission. Vegetables are one of them. A large European study by COMBACTE-CDI explored the contamination rates of retail potatoes in 12 European countries [100]. In the survey, 33 of 147 samples were *C. difficile* positive without notable differences in prevalence between countries. There was a notable difference in the positivity rate between visibly clean potatoes (2/63, 3.2%) and those covered with moderate (14/38, 36.8%) or excessive amounts of soil (17/46, 37%). In this survey, the most common RTs are RT126, RT023, RT010, and RT014/020, in part overlapping the human *C. difficile* isolates [100]. These results are similar to those from a previous survey in Slovenia [101] but lower than those from Australia [102]. Importantly, even when potatoes are washed, peeled and cooked, they can still act as a vector for *C. difficile* spores.

A similar survey in Australia covered more vegetable types and found *C. difficile* in 30/100 samples. Of 43 different isolates, 22 were toxigenic, and many of the RTs were also found in humans and Australian animals [102].

The survey in Slovenia tested 154 different vegetables and got 28 positive results. From those, 115 isolates were obtained and distributed in 25 RTs. Ten RTs were toxigenic, and RT 014/020 was the most common. A study in France tested ready-to-eat salads with raw vegetables and isolated *C. difficile* from three samples (2.9%), two from lettuce-containing salads and one from pea sprouts [103].

The environment is also an important vector. *C. difficile* has been observed in puddle water (14.4%) and soil (36.7%) in a survey from the USA in 2016. The authors observed 34 ribotypes, and most of them were nontoxigenic [104]. Another report showed that *C. difficile* could also be found in environmental samples—from parks (24.6%), followed by homes (17.1%), hospitals (16.5%), commercial stores (8.1%), chain stores (7.6%), and fast-food restaurants (6.5%). An important finding in this report is that the authors observed a similar distribution of ribotypes between clinical, hospital and environmental isolates (RT014/020, 002, 078/126 as most common) with the exception that ribotype 027, which was significantly more common in the clinical isolates compared with the environmental isolates [105]. A recent study detected ST11 (RT078 and RT126) strains in spinach [106]. A very large study covering a wide part of the world (North America (Mexico), Central America (Peru, Guatemala), South America (Brazil), Europe (France, Germany, Italy), and Asia (Taiwan, India)) showed high *C. difficile* contamination rates (~24%), which were similar for healthcare buildings and non-healthcare buildings. The rates were much higher (46.5%) for floor samples [107]. Interestingly, the observed RTs were similar in hospital and non-hospital samples (RT014-020 and RT106 in the first two places, with the exception that in a hospital sample, the authors detected RT027 isolates). Most of the observed RTs were toxigenic [107]. Similar results were reported by authors from the USA. They investigated inside and outside healthcare samples and shoe soles in the USA and 14 other countries. They found 26% of inside and outside healthcare samples to be positive for toxigenic *C. difficile* strains. Shoe soles had the highest positivity rates, with 45% of worldwide samples [108]. 

This wide dissemination of the *C. difficile* spores showed the need for some measures to prevent CDIs. Prevention of CDI includes infection control measures—good sterilisation and disinfection, use of disposable gloves and aprons, and isolation of the CDI patients. Very important is mechanically removing the spores with hand washing with soap and water. Alcohol-based products do not damage the *C. difficile* spores, whereas mechanical hand washing with running water and soap prevents the spread of the spore. Chlorine-based solutions are commonly recommended for environmental decontamination [9].

### 3.4. CDI Treatment

One of the most serious problems with CDI treatment is that antibiotics are both treatment options and risk factors for CDI. That leaves only a few possibilities for treatment. It is worth mentioning that therapy should be started only in patients with CDI symptoms. The standard antibiotic therapy, guided by EUCAST in 2014, consists of metronidazole and vancomycin. Later studies showed the superiority of vancomycin to metronidazole, with clinical success for the primary treatment at 81% versus 73%, respectively [9]. 

The cornerstone in the treatment of CDIs except vancomycin is fidaxomicin. It is a macrocyclic narrow-spectrum antibiotic. It is efficient against *C. difficile*, with no significant influence on the gut microbiota. Fidaxomicin is active, comparable to vancomycin, and can reduce recurrent infections. Recently updated guidelines, defined by the Infectious Diseases Society of America (IDSA) and Society for Healthcare Epidemiology of America (SHEA) in 2017, postulated that fidaxomicin and vancomycin are drugs for the first episode of the infection, independent of its severity. Fidaxomicin gave very low levels of recurrences [109]. For the first recurrence, vancomycin or fidaxomicin are also the only options [9]. Metronidazole is in consideration if it is used in combination with vancomycin. 

It is very important to note that if CDI happens during an antibiotic course, the antibiotic should be discontinued, and if such therapy is indispensable, it should be changed, preferably by antibiotics that are associated with a lower risk of CDI, such as macrolides, aminoglycosides, sulphonamides, vancomycin or tetracyclines [9]. The lack of antibiotic alternatives pushes researchers to evaluate different options. One such option is Bezlotoxumab (a monoclonal antibody that binds to *C. difficile* toxin B), approved by the FDA in 2016 for the prevention of recurrent CDI in patients with a high risk of recurrence. However, its use is limited by the high cost and potential side effects (heart failure) [9]. With very good results is the manipulation of gut microbiota—most often by faecal microbiota transplantation (FMT). Faecal samples from the healthy donor are introduced to CDI patients via oral (capsules) or rectal (colonoscopy) pathways. Antibiotic withdrawal, together with FMT, has 75–90% success and a high rate of prevention of recurrent CDI among all therapeutic options. The first faecal microbiota product has been approved by FDA in the US (Rebyota) [110] and Australia (BIOMICTRA) [111]. The possible problems associated with FMT could be transferring infectious pathogens from the donor to the recipient and the development of autoimmune disorders [9]. 

A newer option is blocking the toxins. Calcium aluminosilicate and human serum albumin have shown toxin-binding activity in vitro; some researchers report that aspirin has a good effect, and recent data has shown that human alpha-defensins and ambroxol have antitoxic activity [112,113]. The information for the important treatment options is shown in Table 1. 

The problem with *C. difficile* became worse after the COVID pandemic. Some studies have reported significant antibiotic overuse in patients with COVID-19, predominantly in hospital settings, and a significant decrease in community antibiotic consumption, especially in 2020 [121,122], with antibiotic prescribing falling 26.8% in 2020 compared to prior years in the USA [123]. It is logical to suppose that hospitalised patients have disrupted gut microbiota and this will increase the number of CDIs, but on the contrary, a strong decrease in *C. difficile* incidence was observed, likely due to improvements in hand hygiene, use of personal protective equipment, and environmental cleaning in the healthcare settings [124]. However, the COVID pandemic has had a big influence; a recent study showed that COVID-19 changed the CDI patterns with a significantly greater percentage of a lethal outcome, 29.5% vs 6.6% [125]. Another study from Canada for 2020/2021 reported an increased frequency of CDIs [126].

## 4. Conclusions

Taking together the high virulence of *C. difficile*, the severe disease that it causes, its higher incidence, and its wide variety of risk factors, which altogether cause increased morbidity and mortality rates, it is not surprising that the CDC has recently reclassified *C. difficile* as an “urgent threat”, but maybe this is not enough, and we should accept this microorganism, especially hypervirulent strains, as “superbugs” which requires increased prevention measures.

The future perspective is the development of live vaccines. For example, researchers evaluated a novel chimeric protein (designated Tcd169), comprised of the glucosyltransferase domain (GT), cysteine protease domain (CPD), and a receptor binding domain (RBD) of TcdB, and the RBD of TcdA. Parenteral immunisations with Tcd169 provided effective protection against infection with the ribotype (RT) 027 in mice [127]. Some new vaccines are investigated on the basis of the S layer [128].

## Figures and Tables

**Table 1:** Important treatment options for *C. difficile* infections [114,115,116,117,118,119,120].

Drug	Mechanisms of Action	Advantages	Disadvantage
	**Standard Antibiotic Therapy**		
Vancomycin	Blocks peptidoglycan synthesis	Superior than metronidazole, can be applied orally and is not absorbed through gut mucosa	Pressure on the intestinal microbiota and risks for recurrence
Fidaxomicin	inhibits bacterial RNA polymerase	Can be used as first-line treatment for primary CDI, reduces recurrence—especially if used in an extended-pulsed regimen	Not superior to vancomycin for RT027 *C. difficile* strains
Metronidazole	Damages DNA in reduced form	Can be used in mild CDIs, low cost	Possibility for recurrence, side effects, such as allergic reactions, gastrointestinal irritation and neuropathy and interactions with ethanol and some common drugs (e.g., warfarin); increase of vancomycin-resistant enterococci
	**Antibiotic Therapy—In Investigation**		
Ridinilazole	Inhibition of cell division (bactericidal) and toxin production	Narrow-spectrum; non-absorbable; high inhibitory activity against *C. difficile* and minimal activity against both Gram-positive and Gram-negative aerobic and anaerobic intestinal microorganisms. Decreases recurrent infections In phase II/III trials, FDA fast-tracking status	More studies are required
Ibezapolstat	DNA polymerase IIIC inhibitor	Activity against *C. difficile* and preservation of Actinobacteria and bile acid equilibrium.	Clinical benefits of the agent should be more widely investigated
	**Toxin-blocking treatment (antibody)**		
Bezlotoxumab	Fully humanised monoclonal antibody against *C. difficile*—blocks TcdB toxin	Good adjunctive to other therapy, decrease recurrent CDIs	Risks for patients with history of heart failure. Parenteral administration, cost
	**Microbiome-modulating therapy**		
Faecal microbiota transplantation (FMT)	Reverts normal balance in gut microbiota	75–90% success, decrease recurrence	FMT is not available everywhere, and all patients are not eligible for the procedure. Long-term safety- the main risk is transfer of infectious pathogens from the donor to the recipient, difficulties in introducing donor faeces, need for repeat transplantation, autoimmunological disorders
Purified *Firmicutes* spores ((SER-109)	Modulates gut microbiota	Reduce the risk of CDI	There are no important side effects, but the investigation is in progress
Live biotherapeutic (a strain of *Bacillus velezensis*) (ADS024)	Modulates gut microbiota; produces proteases that break down the CD toxins	Potential candidate for preventing the recurrence of CDI	Additional in vitro and clinical studies are needed
Quality-controlled stool product (RBX2660)	Modulates gut microbiota	Potential candidate for preventing the recurrence of CDI	
Probiotics- *Saccharomyces boulardii*, *Lactobacillus* spp.	Modulate gut microbiota	Prophylaxix of *C. difficile* infections	Controversial results, not included in the guidelines
